# Clinical and Analytical Performance of the BD Onclarity HPV Assay with SurePath Screening Samples from the Danish Cervical Screening Program Using the VALGENT Framework

**DOI:** 10.1128/JCM.01518-19

**Published:** 2020-01-28

**Authors:** Jesper Hansen Bonde, Helle Pedersen, Wim Quint, Lan Xu, Marc Arbyn, Ditte Møller Ejegod

**Affiliations:** a Department of Pathology, Copenhagen University Hospital, Copenhagen, Denmark; b DDL Diagnostics Laboratory, Rijswijk, The Netherlands; c Unit of Cancer Epidemiology, Belgian Cancer Centre, Sciensano, Brussels, Belgium; University of Iowa College of Medicine

**Keywords:** HPV, HPV genotyping, Onclarity HPV assay, VALGENT, diagnostic test accuracy, test validation

## Abstract

The *Val*idation of HPV *Gen*otyping *T*ests (VALGENT) framework is an international cooperation designed to evaluate human papillomavirus (HPV) assays with genotyping capabilities. Here, we assessed the performance of the BD Onclarity assay using Danish SurePath cervical screening samples collected under the fourth VALGENT installment, consisting of 998 consecutive samples from a screening population and 297 enriched samples with abnormal cytology (100 with atypical squamous cells of undetermined significance [ASCUS], 100 with low-grade squamous intraepithelial lesions [LSIL], and 97 with high-grade squamous intraepithelial lesions [HSIL]). The Onclarity assay detects six HPV genotypes individually (genotypes 16, 18, 31, 45, 51, and 52) and eight genotypes in three bulks (genotypes 33 and 58; genotypes 56, 59, and 66; and genotypes 35, 39, and 68). The clinical performance of the Onclarity assay for the detection of cervical intraepithelial neoplasia of grade 2 or worse (≥CIN2) and of two consecutive cytology outcomes negative for intraepithelial lesion or malignancy (2×NILM) was assessed relative to that of the GP5+/6+ PCR-enzyme immunoassay (GP-EIA) by a noninferiority test. The relative sensitivity for ≥CIN2 was 1.00 (95% confidence interval [CI], 0.97 to 1.04), and the relative specificity for 2×NILM was 1.04 (95% CI, 1.02 to 1.06). The Onclarity assay was found to be noninferior to the GP-EIA in terms of both sensitivity (*P* = 0.0006) and specificity (*P* < 0.0001). The type-specific performance of the Onclarity assay was also assessed, using the GP5+/6+ PCR with Luminex genotyping (GP-LMNX) as the comparator. The Onclarity assay showed good concordance for almost all HPV genotype groups. A stability analysis of SurePath samples was also performed, where a SurePath aliquot was stored refrigerated for 7 months and the internal control of the Onclarity assay was used as a marker for cellularity. The threshold cycle (*C_T_*) value was the same (24.8) in the first and second Onclarity runs, showing that a SurePath sample can be stored refrigerated for 7 months and still remain a valid test specimen.

## Abstract (precis)

The *Val*idation of HPV *Gen*otyping *T*ests (VALGENT) framework is an international cooperation designed to evaluate human papillomavirus (HPV) assays with genotyping capabilities.

## INTRODUCTION

Careful clinical validation of human papillomavirus (HPV) assays is increasingly important as primary HPV screening is replacing cytology-based cervical cancer screening. The clinical performance of any HPV test in cervical screening relies on its ability to detect infections associated with cervical intraepithelial neoplasia of grade 2 or worse (≥CIN2) without overdiagnosing clinically irrelevant HPV infections. In 2009, Meijer et al. established guidelines and requirements for clinical sensitivity, specificity, and reproducibility with which novel HPV tests must comply in order to be used for primary clinical screening (1). Compared to the number of assays that are commercially available today, a surprisingly small number of HPV assays are clinically validated with the international criterion (2–4). HPV assay development over the past decade has led to a host of PCR-based assays reporting high-risk HPV (hrHPV) findings with various degrees of data resolution. In this regard, HPV assays can be stratified into four categories: (i) consensus assays that report only positive or negative outcomes resulting from measurement of the presence of the 13 or 14 most common oncogenic HPV genotypes (genotypes 16, 18, 31, 33, 35, 39, 45, 51, 52, 56, 58, 59, 68, and sometimes 66); (ii) consensus assays with limited genotype reporting, often for HPV16 and HPV18; (iii) HPV assays with extended genotyping, typically identifying HPV16 and -18 combined with more, but not all, of the oncogenic genotypes; and (iv) full genotyping assays with individual reporting of 14 oncogenic HPV genotypes. The Hybrid Capture 2 assay (HC2; Qiagen, Hilden, Germany) and GP5+/6+ PCR-enzyme immunoassay (GP-EIA; DDL Diagnostic Laboratory, Rijswijk, The Netherlands) have been considered standard comparator assays, since both have demonstrated longitudinal evidence of protection against cervical precancer and cancer through randomized trials (5, 6). The international validation criterion evaluates assay performance against one of these two standard comparator assays (1) for combined detection of 13 or 14 hrHPV genotypes but does not allow for more-advanced performance evaluation at the level of individual hrHPV genotypes detected by an assay. Given the development of technology in the field of HPV screening and diagnostics since 2009, this represents a limitation of the original international guidelines. As more HPV assays with various degrees of genotype detection capabilities become commercially available (1), it is imperative that assay performance be assessed robustly with well-annotated cervical samples that are representative for a screening population.

The VALGENT (*Val*idation of HPV *Gen*otyping *T*ests) framework represents an international collaboration designed to evaluate the comparative performance of HPV assays with genotyping capacity for use in primary cervical cancer screening (7–13). The VALGENT validation panels take into account different sample collection media and include samples from women attending routine screening as well as a disease-enriched group with cytologically defined abnormal samples (7, 8). In order to allow comparison with other HPV assays, each VALGENT panel includes a comparator assay that is clinically validated for cervical screening. The detailed objectives and study design of the fourth installment of the VALGENT framework (VALGENT4) and previous VALGENT panels have been published elsewhere (7, 8).

To date, four VALGENT panels have been collected from the Belgian (VALGENT1) (9–11), Scottish (VALGENT2) (12–15), Slovenian (VALGENT3) (16–20), and Danish (VALGENT4) (8) cervical cancer screening programs. VALGENT4 specifically comprises a panel of samples collected in SurePath medium; previously, the majority of clinically validated HPV assays for use in screening had been undertaken on samples collected with the ThinPrep system.

Here, we present the clinical validation of the BD Onclarity HPV assay (BD Diagnostics, Sparks, MD, USA) using Danish SurePath cervical screening samples from the fourth installment of the VALGENT framework. The Onclarity assay is an extended genotyping assay providing individual detection of six HPV genotypes (genotypes 16, 18, 31, 45, 51, and 52) and the identification of eight additional genotypes in three bulks (genotypes 33 and 58 [33/58], 56/59/66, and 35/39/68). The validation was performed using the international guidelines, with the GP-EIA as a comparator for clinical evaluation. In addition, the stability of stored SurePath cervical samples for HPV analysis was assessed over a 7-month period.

## MATERIALS AND METHODS

### Sample collection and histological follow-up.

The sample collection process has been described in detail elsewhere (8). Briefly, the VALGENT4 panel was collected from women participating in the Danish cervical cancer screening program at the Department of Pathology, Hvidovre Hospital, Hvidovre, Denmark (the parent laboratory). The VALGENT panel is standardized (7), here comprising 998 consecutive screening samples from the routinely screened Danish population (the screening population) as well as a disease-enriched component with cytological abnormalities (100 samples with atypical squamous cells of undetermined significance [ASCUS], 100 with low-grade squamous intraepithelial lesions [LSIL], and 97 with high-grade squamous intraepithelial lesions [HSIL]) (the enriched population). All samples were collected in SurePath medium. All cytology and histology procedures were performed at the parent laboratory as described previously (8), all clinical follow-up was managed according to the Danish guidelines, and the outcome of the VALGENT4 HPV testing did not affect the clinical outcome assessment. Subsequent histological follow-up, if any, on women included in the VALGENT4 study was retrieved from the Danish PatoBank.

**FIG 1:**
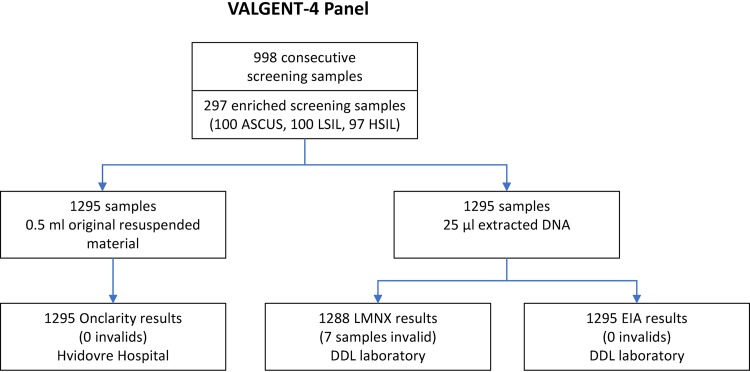
Flow chart for collection of the VALGENT4 panel and HPV testing. ASC-US, atypical squamous cells of undetermined significance; LSIL, low-grade squamous intraepithelial lesions; HSIL, high-grade squamous intraepithelial lesions.

A flow chart of the procedure for sample collection and HPV testing is shown in Fig. 1.

### Comparator assay testing.

DNA was extracted from the VALGENT4 panel samples as described previously (8) and was shipped to the DDL Diagnostics Laboratory (Rijswijk, The Netherlands), where all GP5+/6+ PCR testing was performed. The clinically validated hrHPV GP-EIA for pooled detection of 14 oncogenic types (genotypes 16, 18, 31, 33, 35, 39, 45, 51, 52, 56, 58, 59, 66, and 68) was used as a comparator for the clinical performance of the Onclarity assay. For genotype concordance analysis, a Luminex-based readout was used (GP-LMNX) for individual genotyping of the 14 HPV types (14). The mean time from sample reception to DNA extraction was 27 days (range, 11 to 71 days). GP-LMNX testing on biobanked DNA aliquots was completed 685 days after sample reception. GP-EIA testing was subsequently performed and completed on the GP5+/6+ amplicons 1,008 days after sample reception. The samples were stored refrigerated.

### The BD Onclarity HPV assay.

The Onclarity HPV assay is a real-time PCR DNA assay, targeting the E6 and E7 DNA regions of the HPV genome, which detects 14 oncogenic HPV genotypes in nine genotype readouts (genotypes 16, 18, 31, 45, 51, 52, 33/58, 56/59/66, and 35/39/68). The assay harbors an internal human β-globin (HBB) control for sample adequacy and assay performance. A specimen was considered adequate when the HBB threshold cycle (*C_T_*) value was <34.2 or any hrHPV could be identified. The full Onclarity assay workflow on the automated Viper Lt platform has been described in detail previously (21). Briefly, 0.5 ml of original resuspended SurePath material was transferred to a BD tube containing 1.7 ml cervical brush diluent (CBD) medium. The samples were prewarmed at 120°C for 30 min prior to being transferred to the fully automated Viper Lt platform, where the samples were tested with the Onclarity HPV assay according to the manufacturer’s recommendations. All Onclarity testing was performed at the parent laboratory. The mean time from sample reception at the laboratory to Onclarity testing was 28 days (range, 2 to 70 days). The samples were stored refrigerated prior to testing.

### Stability of SurePath-collected cervical samples for analysis of HPV.

In addition to the baseline Onclarity analysis, 0.5 ml of SurePath material from 1,212 samples was aliquoted into a separate Eppendorf tube and refrigerated (4°C) for 7 months until testing with the Onclarity assay on the Viper Lt platform. Eighty-five samples did not have enough material for the stability aliquot. The stability and cellularity of the VALGENT4 panel were assessed using values from each individual sample’s internal control of the Onclarity assay as markers for cellularity and analytical stability over time. The Onclarity assay has a three-well design with nine genotype readouts, and the internal HBB control is included in each well.

### Data analysis.

A sample was considered Onclarity positive for one of the nine genotype groups if the *C_T_* value was below 34.2, according to the manufacturer’s recommendations. For the GP-EIA and the GP-LMNX assay, a sample was reported positive if at least one of the 14 genotypes reported by the Onclarity assay was detected. The level of genotype agreement between the Onclarity and GP-LMNX assays was determined by using kappa statistics. In addition, if a sample was GP-LMNX positive for HPV33 and HPV58, the infection counted only once in the genotype 33/58 pool; the same was true for the HPV56/59/66 and HPV35/39/68 pools.

For clinical validation, women with confirmed cervical intraepithelial neoplasia of grade 2 or worse (≥CIN2), ≥CIN3, or cervical cancer within 33 months (range, 32 to 35 months) after sample collection were classified as having high-grade disease (the diseased population). Women with two consecutive NILM (negative for intraepithelial lesion or malignancy) cytology outcomes at enrollment and at 12 to 24 months prior were classified as having no evidence of disease (the 2×NILM control population). The accuracy of the Onclarity assay for the detection of ≥CIN2 and ≥CIN3 was assessed and compared to that of the GP-EIA, which was used as the comparator for clinical performance. The Onclarity assay was assessed statistically for noninferiority to the GP-EIA with a score test for matched proportions, using 0.90 and 0.98 as benchmarks for relative sensitivity and specificity, respectively (1, 22).

The results of testing by the Onclarity assay, GP-EIA, and GP-LMNX assay were sent to the Unit of Cancer Epidemiology, Sciensano, Brussels, Belgium, for statistical analysis, which was performed using STATA, version 14 (StataCorp, College Station, TX, USA).

For stability analysis, the *C_T_* value of the HBB was calculated as an average of the three individual HBB *C_T_* values for the baseline test and was compared with that for the 7-month endpoint test.

### Ethical approval.

Sample collection and data retrieval for VALGENT4 are approved by the Danish Data Inspection Agency (J. no. AHH-2017-024; I-Suite 05356). An EU General Data Protection Regulation (GDPR)-compliant data handler agreement was established between the principal site, Copenhagen University Hospital, Copenhagen, Denmark, and Sciensano, Brussels, Belgium, for the data analysis. All samples collected were cross-referenced and found eligible with the Danish register relating to the collection, storage, and use of human biological material in health research projects (Vævsanvendelsesregistret).

## RESULTS

### Demographic, pathological, and HPV characteristics of the VALGENT4 panel.

When the entire VALGENT4 cohort (the screening and enriched populations together) was considered, the average age was 42.2 years (range, 30 to 59 years). The cytological stratification of the screening population was as follows: 947 were NILM, 6 had ASCUS, 21 had LSIL, and 24 had either HSIL, atypical glandular cells (AGC), atypical squamous cells for which HSIL could not be excluded (ASCH), or adenocarcinoma *in situ* (AIS) (average age, 42.8 years [range, 30 to 59 years]) (Table 1). In the enriched population, the average age was 40.4 years (range, 30 to 59 years). Histological follow-up retrieval revealed 122 women with ≥CIN2; the majority of the ≥CIN2 cases were derived from the enriched population (*n* = 109). A total of 897 women had two consecutive NILM cytology smears (2×NILM), which were used for the specificity calculations.

**TABLE 1:** Characteristics of the study population of the VALGENT4 panel and prevalence of hrHPV as assessed by the Onclarity assay and the GP-EIA

Characteristic^*a*^	Total	No. (%) hrHPV positive by the:	
		Onclarity assay	GP-EIA
Group			
All	1,295	368 (28.4)	396 (30.6)
Screening	998	113 (11.3)	143 (14.3)
Enriched	297	255 (85.9)	253 (85.2)
Age (yr)			
30–39	531	192 (36.2)	202 (38.0)
40–49	519	126 (24.3)	136 (26.2)
50–59	245	50 (20.4)	58 (23.7)
Cytology			
Normal	947	73 (7.7)	105 (11.1)
ASCUS	106	103 (97.2)	97 (91.5)
LSIL	121	88 (72.7)	88 (72.7)
HSIL	106	93 (87.7)	96 (90.6)
AGC/ASC-H/AIS	15	1 (73.3)	0 (66.7)
Finding upon histological follow-up			
No follow-up	946	106 (11.2)	139 (14.7)
CIN0	154	82 (53.2)	78 (50.6)
CIN1	73	67 (91.8)	66 (90.4)
CIN2	39	33 (84.6)	35 (89.7)
CIN3	75	72 (96.0)	70 (93.3)
Cancer	8	8 (100)	8 (100)
≥CIN2	122	113 (92.6)	113 (92.6)
2×NILM	897	66 (7.4)	97 (10.8)

a ASCUS, atypical squamous cells of undetermined significance; LSIL, low-grade squamous intraepithelial lesions; HSIL, high-grade squamous intraepithelial lesions; AGC, atypical glandular cells; ASC-H, atypical squamous cells for which HSIL cannot be excluded; AIS, adenocarcinoma *in situ*; CIN, cervical intraepithelial neoplasia; ≥CIN2, CIN of grade 2 or worse; NILM, negative for intraepithelial lesions or malignancies; 2×NILM, NILM at both the prior screening round and the index screening.

In the screening and enriched populations, the Onclarity assay showed hrHPV prevalences of 11.3% and 85.9%, respectively (28.4% in the total population) (Table 1). In comparison, the GP-EIA showed prevalences of 14.3% and 85.2% in the screening and enriched populations, respectively (31.7% in the total population). The rate of HPV positivity in the screening population by the Onclarity assay was highest for women aged 30 to 39 years (16.4%) and lower in women aged 40 to 49 (7.6%) or 50 to 59 (9.2%) years (Table 2). The prevalences of HPV genotypes 16, 52, 31, 18, 45, and 51 in the screening population were 1.8%, 1.7%, 1.2%, 1.1%, 1.1%, and 0.8%, respectively (given in decreasing order) (Table 2). The prevalences of the three bulk genotype groups were 2.3% for HPV56/59/66, 2.3% for HPV35/39/68, and 1.7% for HPV33/58 (Table 2). The GP-LMNX assay had the highest prevalence for seven of nine HPV genotype groups in the normal cytology samples, whereas the Onclarity assay had the highest overall HPV prevalence in ASCUS, LSIL, and HSIL samples for most genotype groups (Table 3).

**TABLE 2:** Prevalences of HPV genotypes by age in the screening population as assessed by the Onclarity assay

HPV type	No. (%) positive^*a*^ in the following age group (yr):			No. (%) positive in the total population (*n* = 998)
	30–39 (*n* = 383)	40–49 (*n* = 408)	50–59 (*n* = 207)	
All hrHPV types	63 (16.4)	31 (7.6)	19 (9.2)	113 (11.3)
HPV16	10 (2.6)	5 (1.2)	3 (1.4)	18 (1.8)
HPV18	7 (1.8)	4 (1.0)	0 (0.0)	11 (1.1)
HPV31	6 (1.6)	5 (1.2)	1 (0.5)	12 (1.2)
HPV45	6 (1.6)	3 (0.7)	2 (1.0)	11 (1.1)
HPV51	4 (1.0)	2 (0.5)	2 (1.0)	8 (0.8)
HPV52	12 (3.1)	1 (0.2)	4 (1.9)	17 (1.7)
HPV33/58	8 (2.1)	4 (1.0)	5 (2.4)	17 (1.7)
HPV56/59/66	14 (3.7)	6 (1.5)	3 (1.4)	23 (2.3)
HPV35/39/68	13 (3.4)	7 (1.7)	3 (1.4)	23 (2.3)

a All infections observed were counted regardless of whether they were observed as single infections or multiple infections.

**TABLE 3:** HPV genotyping prevalences according to the Onclarity and GP-LMNX assays, stratified by cytology result

Assay and HPV genotype	No. (%) of samples with the following cytology result^*a*^:					Prevalence (no. [%]) among all samples (*n*, 1,295 or 1,288)^*b*^
	Normal (*n*, 947 or 940)^*b*^	ASCUS (*n*, 106)	LSIL (*n*, 121)	HSIL (*n*, 106)	AGC/ASC-H/AIS (*n*, 15)	
Onclarity assay						
All hrHPV types	73 (7.7)	103 (97.2)	88 (72.7)	93 (87.7)	11 (73.3)	368 (28.4)
HPV16	12 (1.3)	26 (24.5)	12 (9.9)	36 (34.0)	2 (13.3)	88 (6.8)
HPV18	6 (0.6)	7 (6.6)	6 (5.0)	10 (9.4)	2 (13.3)	31 (2.4)
HPV31	7 (0.7)	13 (12.3)	13 (10.7)	16 (15.1)	1 (6.7)	50 (3.9)
HPV45	7 (0.7)	13 (12.3)	5 (4.1)	8 (7.5)	2 (13.3)	35 (2.7)
HPV51	3 (0.3)	7 (6.6)	11 (9.1)	8 (7.5)	1 (6.7)	30 (2.3)
HPV52	13 (1.4)	12 (11.3)	12 (9.9)	11 (10.4)	0 (0)	48 (3.7)
HPV33/58	10 (1.1)	13 (12.3)	7 (5.8)	14 (13.2)	2 (13.3)	46 (3.6)
HPV56/59/66	12 (1.3)	20 (18.9)	37 (30.6)	10 (9.4)	3 (20.0)	82 (6.3)
HPV35/39/68	18 (1.9)	19 (17.9)	18 (14.9)	5 (4.7)	1 (6.7)	61 (4.7)
GP-LMNX assay						
All hrHPV types	122 (13.0)	97 (91.5)	86 (71.1)	97 (91.5)	10 (66.7)	412 (32.0)
HPV16	25 (2.7)	26 (24.5)	11 (9.1)	38 (35.8)	2 (13.3)	102 (7.9)
HPV18	23 (2.4)	8 (7.5)	7 (5.8)	10 (9.4)	2 (13.3)	50 (3.9)
HPV31	11 (1.2)	12 (11.3)	12 (9.9)	16 (15.1)	1 (6.7)	52 (4.0)
HPV45	15 (1.6)	14 (13.2)	5 (4.1)	7 (6.6)	2 (13.3)	43 (3.3)
HPV51	9 (1.0)	8 (7.5)	12 (9.9)	8 (7.5)	0 (0)	37 (2.9)
HPV52	9 (1.0)	9 (8.5)	8 (6.6)	6 (5.7)	0 (0)	32 (2.5)
HPV33/58^*c*^	21 (2.2)	13 (12.3)	8 (6.6)	13 (12.3)	3 (20.0)	58 (4.5)
HPV56/59/66^*c*^	23 (2.4)	22 (20.8)	37 (30.6)	13 (12.3)	3 (20.0)	98 (7.6)
HPV35/39/68^*c*^	17 (1.8)	15 (14.2)	13 (10.7)	3 (2.8)	1 (6.7)	49 (3.8)

a ASCUS, atypical squamous cells of undetermined significance; LSIL, low-grade squamous intraepithelial lesions; HSIL, high-grade squamous intraepithelial lesions; AGC, atypical glandular cells; ASC-H, atypical squamous cells for which HSIL cannot be excluded; AIS, adenocarcinoma *in situ*.

b Seven samples were invalid with the GP-LMNX assay.

c HPV genotypes have been pooled for comparison with the Onclarity assay. An HPV33-positive result, therefore, is counted in a combined HPV33/58 outcome. A multiple infection with, e.g., HPV33 and HPV58 is counted only once, as an HPV33/58 infection. The same applies to testing of HPV56/59/66 and HPV35/39/68.

### Clinical performance of the Onclarity assay.

Table 4 presents the cross-tabulations of the results of the Onclarity assay and the comparator assay, the GP-EIA, for women with ≥CIN2, ≥CIN3, and 2×NILM. Table 5 shows the sensitivities of the Onclarity assay and the GP-EIA for ≥CIN2 and ≥CIN3 cases and the specificities of these assays for 2×NILM subjects, as well as the relative sensitivities and specificity of the Onclarity assay. The Onclarity assay reported 113 of the 122 ≥CIN2 cases to be hrHPV positive (sensitivity, 92.6% [95% confidence interval {CI}, 86.5 to 96.6%]); in comparison, the GP-EIA also detected hrHPV in 113 of 122 cases (sensitivity, 92.6% [CI, 86.5 to 96.6%]). The relative ≥CIN2 sensitivity of the Onclarity assay was 1.00 (CI, 0.97 to 1.04). The Onclarity assay detected 80 of 83 ≥CIN3 cases (sensitivity, 96.4% [CI, 89.9 to 99.2%]), and the GP-EIA detected 78 of 83 cases (sensitivity, 94.0% [CI, 86.5 to 98.0%]). The relative sensitivity of the Onclarity assay for ≥CIN3 was 1.03 (CI, 0.99 to 1.06). The Onclarity assay was found to be noninferior to the GP-EIA for ≥CIN2 (*P* = 0.0006) and ≥CIN3 (*P* < 0.0001) sensitivity. A total of 897 2×NILM cases were found; the Onclarity assay called 831 of them negative (specificity, 92.6% [CI, 90.7 to 94.3%]), while the GP-EIA found 800 of 897 to be negative (specificity, 89.2% [CI, 87.0 to 91.1%]). The relative specificity of the Onclarity assay was 1.04 (CI, 1.02 to 1.06). The Onclarity assay was found to be noninferior to the GP-EIA for specificity (*P* < 0.0001).

**TABLE 4:** Comparison of results of the Onclarity assay and the GP-EIA for ≥CIN2, ≥CIN3, and <CIN1 populations

Study population^*a*^ (no.) and Onclarity assay result	GP-EIA result (no.)		Total
	Positive	Negative	
≥CIN2 (122)			
Positive	111	2	113
Negative	2	7	9
Total	113	9	122
≥CIN3 (83)			
Positive	78	2	80
Negative	0	3	3
Total	78	5	83
2×NILM (897)			
Positive	60	6	66
Negative	37	794	831
Total	97	800	897

a CIN, cervical intraepithelial neoplasia; ≥CIN2, CIN of grade 2 or worse; ≥CIN3, CIN of grade 3 or worse; NILM, negative for intraepithelial lesions or malignancies; 2×NILM, NILM at both the prior screening round and the index screening.

**TABLE 5:** Clinical accuracy of the Onclarity assay and the GP-EIA^*a*^ for ≥CIN2, ≥CIN3, and <CIN1 outcomes

Outcome (no.)	Measure	% absolute accuracy (95% CI)		Relative accuracy of Onclarity assay vs GP-EIA (95% CI)	*P* value for noninferiority test^*b*^
		Onclarity assay	GP-EIA		
≥CIN2 (122)	Sensitivity	92.6 (86.5–96.6)	92.6 (86.5–96.6)	1.00 (0.97–1.04)	0.0006
≥CIN3 (83)	Sensitivity	96.4 (89.9–99.2)	94.0 (86.5–98.0)	1.03 (0.99–1.06)	<0.0001
2×NILM (897)	Specificity	92.6 (90.7–94.3)	89.2 (87.0–91.1)	1.04 (1.02–1.06)	<0.0001

a The GP-EIA was used as a comparator test.

b A *P* value of <0.05 for the noninferiority test means that the sensitivity or specificity of the Onclarity assay is not significantly lower than that of the GP-EIA, using the benchmarks of 0.90 and 0.98 for relative sensitivity and relative specificity, respectively.

### HPV genotyping concordance between the Onclarity and GP-LMNX assays.

The hrHPV agreement between the Onclarity and GP-LMNX assays was 93.6% (kappa, 0.85) for the total VALGENT4 population; for the screening population, the agreement was 93.1% (kappa, 0.71), and for the enriched population, it was 95.3% (kappa, 0.81) (Table 6). The genotype concordance in the whole VALGENT population was good (kappa, 0.73 to 0.80) to excellent (kappa, >0.80), with kappa values ranging from 0.73 to 0.90. When genotype detection by the Onclarity and GP-LMNX assays was stratified by VALGENT4 subsets, the concordance was better for the enriched population, with a kappa range from 0.79 to 0.98, than for the screening population (kappa range, 0.56 to 0.86). The kappa values for almost all genotype groups were higher in the enriched population than in the screening population. Only for HPV18 was the concordance below 0.6, and this was limited to the screening population.

**TABLE 6:** Detection of individual oncogenic genotypes by the Onclarity and GP-LMNX assays in the screening and enriched populations of the VALGENT4 panel

hrHPV genotype(s)	Screening population (*n* = 991)								Enriched population (*n* = 297)								VALGENT4 panel	
	No. (%) with the following result(s)^*a*^:						Agreement	Kappa	No. (%) with the following result(s)^*a*^:						Agreement	Kappa		
	Onc+	GP-LMNX+	Onc+, GP-LMNX+	Onc+, GP-LMNX–	Onc–, GP-LMNX+	Onc–, GP-LMNX–			Onc+	GP-LMNX+	Onc+, GP-LMNX+	Onc+, GP-LMNX–	Onc–, GP-LMNX+	Onc–, GP-LMNX–			Agreement	Kappa
HPV16	18 (1.8)	31 (3.1)	18	0	13	960	98.7	0.73	70 (23.6)	71 (23.9)	68	2	3	224	98.3	0.95	98.6	0.90
HPV18	11 (1.1)	28 (2.8)	11	0	17	963	98.3	0.56	20 (6.7)	22 (7.4)	19	1	3	274	98.7	0.90	98.4	0.73
HPV31	12 (1.2)	16 (1.6)	12	0	4	975	99.6	0.86	38 (12.8)	36 (12.1)	35	3	1	258	98.7	0.94	99.4	0.92
HPV45	11 (1.1)	18 (1.8)	9	2	9	971	98.9	0.62	24 (8.1)	25 (8.4)	24	0	1	272	99.7	0.98	99.1	0.84
HPV51	8 (0.8)	13 (1.3)	7	1	6	977	99.3	0.66	22 (7.4)	24 (8.1)	21	1	3	272	98.7	0.91	99.2	0.83
HPV52	17 (1.7)	11 (1.1)	11	6	0	974	99.4	0.78	31 (10.4)	21 (7.1)	21	10	0	266	96.6	0.79	98.8	0.79
HPV33/58	17 (1.7)	29 (2.9)	16	1	13	961	98.6	0.69	29 (9.8)	29 (9.8)	27	2	2	266	98.7	0.92	98.6	0.82
HPV56/59/66	23 (2.3)	20 (2.0)	16	7	4	964	98.9	0.74	38 (12.8)	29 (9.8)	28	10	1	258	96.3	0.82	98.3	0.79
HPV35/39/68	23 (2.3)	33 (3.3)	21	2	12	956	98.6	0.74	59 (19.9)	65 (21.9)	55	4	10	228	95.3	0.86	97.8	0.83
All 14 hrHPV genotypes	113 (11.4)	159 (16.0)	102	11	57	821	93.1	0.71	255 (85.9)	253 (85.2)	247	8	6	36	95.3	0.81	93.6	0.85

a Onc, Onclarity assay; +, positive; –, negative.

### Analytical stability of SurePath screening samples.

**FIG 2:**
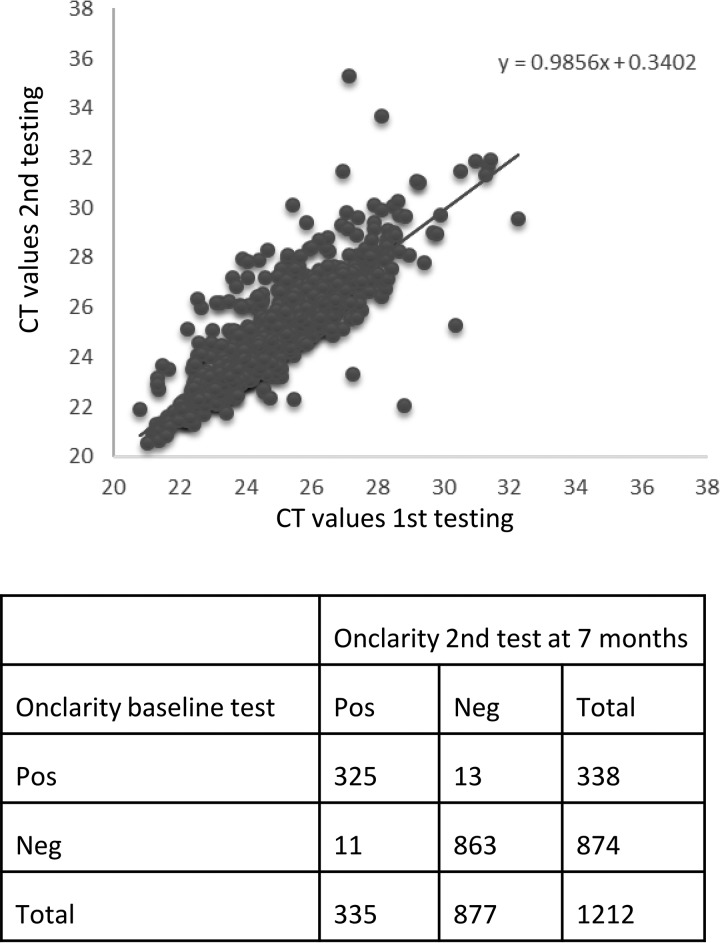
Stability of SurePath cervical screening samples tested on the Onclarity system. (Top) Scatter plot over average HBB *C_T_* values for first testing (baseline) and second testing (at 7 months). (Bottom) Overall concordance between first testing (baseline) and second testing on 1,212 samples.

Of the 1,295 samples, a total of 1,212 had sufficient material for stability testing. Aliquots for stability testing were stored refrigerated for 7 months after baseline testing prior to a second test. Cytology results for the 1,212 samples included were NILM (*n* = 894) and ≥ASCUS (*n* = 318). In total, the results for 1,188 of 1,212 samples (98.0% [CI, 97.0 to 98.7%]) were reproducible after retesting at 7 months (Fig. 2). Of 24 discordant samples, 13 went from HPV positive to HPV negative, whereas 11 samples went from HPV negative to HPV positive, after the second test. Furthermore, the mean *C_T_* values of the internal control, HBB, were similar for the baseline test (mean *C_T_*, 24.8 [CI, 24.71 to 24.89]) and the second test at 7 months (mean *C_T_*, 24.8 [CI, 24.70 to 24.91]) (*P* = 0.96). No differences in *C_T_* values for the detection of individual or bulk genotypes were observed between the first and second tests (data not shown).

## DISCUSSION

In the present analysis, the clinical and type-specific performance of the Onclarity HPV assay was assessed. The Onclarity assay has the ability to detect six individual oncogenic HPV genotypes, whereas the remaining eight oncogenic HPV genotypes are detected in three groups. The Onclarity assay showed similar clinical sensitivity (relative sensitivity, 1.00 [CI, 0.97 to 1.04]) and slightly better specificity (relative specificity, 1.04 [CI, 1.02 to 1.06]) than the standard comparator assay, the GP-EIA, for the detection of ≥CIN2. The Onclarity assay was shown to be noninferior to the comparator assay, the GP-EIA, for both sensitivity (*P* = 0.0006) and specificity (*P* < 0.0001). The hrHPV concordance between the Onclarity and GP-LMNX assays was high when assessed at the level of 14 oncogenic HPV genotypes combined, as well as for all 9 genotype groups (Table 6). The concordance was higher when only the disease-enriched population rather than the screening population subset was considered, both for overall oncogenic HPV detection and at the individual genotype level (Table 6). This finding is similar to those of previous studies looking at concordance between HPV assays, showing that HPV assays have better agreement in samples from women with disease than in screening samples (23, 24).

Some of the discordance observed can be explained on the basis of the assay technologies. The Onclarity HPV assay is a DNA assay targeting the E6 and E7 genes and has an amplification target range from 79 to 137 bp, whereas the GP-EIA and the GP-LMNX assay target the L1 gene and have a target amplification of 150 bp. Furthermore, the Onclarity assay is a real-time PCR assay, whereas the GP-LMNX assay is a PCR-based assay with subsequent Luminex detection. Together, these assay specification differences can to some extent impact detection, especially in samples with a viral load close to the individual assay cutoff between positive and negative.

The Onclarity assay has been validated previously using the VALGENT2 panel, which consisted of ThinPrep screening samples from the Scottish cervical cancer screening program (7, 13); here, the GP-EIA was also used as a comparator (13). In VALGENT2, the Onclarity assay was shown to be noninferior to the GP-EIA for sensitivity (*P* = 0.001) but not for specificity (*P* = 0.186). Cuschieri et al. (13) found that the Onclarity assay detected more infections than the GP-EIA but not significantly more ≥CIN2 cases. This was not observed in our study, where the Onclarity assay detected fewer HPV infections than the GP-EIA and an equal number of ≥CIN2 cases. Cuschieri et al. noted that the VALGENT2 panel included samples from women below the age of 30 years and speculated that the high HPV prevalence in the Scottish population (18%) could negatively impact specificity calculations. For comparison, we included only samples from women ≥30 years old, and the hrHPV prevalence of the screening population subset of the Danish VALGENT4 panel was 11%.

The Onclarity assay has also been validated using the international guidelines (1) with both ThinPrep (25)- and SurePath (21)-collected screening samples. In both studies, the Onclarity assay fulfilled the international guidelines for both specificity and sensitivity and included only samples from women who were 30 years old or older (21, 25).

In this study, a more detailed analysis of individual genotype detection by the Onclarity and GP-LMNX assays showed good concordance for all genotype groups (Table 6) except HPV18, -45, and -51 in the screening population subset, where the GP-LMNX assay detected more infections than the Onclarity assay. For the enriched population subset, the concordance was higher for all genotypes, with the lowest concordance observed for HPV52, HPV56/66/68, and HPV35/39/68. Here, the Onclarity assay detected more HPV52 and HPV56/59/66 infections and fewer HPV35/39/68 infections than the GP-LMNX assay (Table 6). In addition, the GP-LMNX assay detected more HPV16 and -18 infections in NILM cytology samples than the Onclarity assay (Table 3).

The clinical importance of these observations is the demonstration of the ability of the Onclarity assay to precisely detect clinically relevant HPV infections at the individual genotype level. This information can be used with advanced screening algorithms where individual follow-up recommendations are issued for women with HPV16 and -18 found in screening samples or, as recently proposed by us (26) and shortly to be adopted in the Danish screening program, where individual screening recommendations are issued for samples positive for HPV16, -18, -31, -33, and -51, which pose an overall higher risk of ≥CIN2 than the remaining hrHPV genotypes (27, 28).

In this study, we also evaluated the analytical stability of SurePath cervical screening samples for HPV testing and the reproducibility of sample adequacy for the Onclarity assay after storage. This information is relevant because cervical screening samples are typically collected at one site and shipped to a laboratory for analysis; the time frame between those events is defined by geography and logistic infrastructure. Moreover, the ability to reproduce a baseline test outcome is also relevant for quality procedures, where samples can be tested after a period of time, i.e., for quality control or audit purposes. The adequacy of the SurePath liquid-based cytology (LBC) test for molecular HPV analysis has been questioned in the past on the premise that the medium contains a low concentration of formaldehyde in addition to the alcohol fixative to ensure adequate preservation of the cells for cytology evaluation. The cause of concern is the formalin, which induces cross-links between DNA and protein (29–31). The analytical stability of SurePath and Onclarity samples and the reproducibility of results were tested on an aliquot of the VALGENT4 samples stored at 4°C for 7 months after baseline testing. The 7-month period chosen greatly exceeds the time for which most routine laboratories will retain a sample for quality assurance purposes. Our argument is that if stability can be proven for both human and HPV genomic material for such an extended period, discussions regarding the stability of SurePath-collected samples intended for molecular HPV screening can cease. Here, the data show clearly that the stored SurePath samples were analytically stable, and the results clinically reproducible, by Onclarity HPV assay testing after 7 months of storage at 4°C. Using the internal HBB control as a marker for analytical stability and cellularity, the mean *C_T_* at baseline was 24.8 (standard deviation, 1.6) versus 24.8 (standard deviation, 1.8) after storage. The overall reproducibility of baseline test results was 98.0%, with 1,188 of 1,212 samples returning the same test result. Equally important, looking at the genotype outcomes reported, the baseline and poststorage results showed no statistical differences either (data not shown). The international validation of the Onclarity assay on the SurePath and ThinPrep systems previously reported the hrHPV intralaboratory reproducibilities of the Onclarity assay as 97.4% (21) and 98.6% (25), respectively. Supported further by the conclusions of Agreda et al. (32), we conclude that long-term storage of SurePath screening samples poses no analytical issue for HPV testing for at least 7 months of storage in combination with the Onclarity HPV assay.

The strength of this study was that the samples were freshly collected cervical cancer screening samples from women 30 to 59 years old participating in the organized Danish national screening program. Onclarity HPV testing was done within weeks of sample collection (mean, 4 weeks; range, 2 to 70 days). A weakness discussed in the published protocol of VALGENT4 (8) is the sample preparation protocol and aliquoting procedure, which are off-label for the Onclarity assay, GP-EIA, and GP-LMNX assay. However, extensive quality assurance analysis showed that the resuspended samples contained sufficient material for testing, which is reinforced by the observation that few samples were found assay invalid. The number of invalid results by the GP-LMNX assay (rate, 0.54%) was not higher than those observed in the previous VALGENT panels, with invalid rates from 0.25% to 1.9% (13, 17, 18, 20). The relatively short follow-up period (maximum, 3 years) in VALGENT4 is a limitation for interpretation of the long-term safety of a negative screening result. However, since the hrHPV GP-EIA originally was validated through randomized trials with follow-up currently reported at 14 years, the cross-sectional accuracy of this comparator test is well acknowledged for validation studies. The origin of the VALGENT4 panels allows for the retrieval of further follow-up data from the Danish PatoBank at a later point in time, to provide information on long-term safety.

In conclusion, the Onclarity assay has high sensitivity for the detection of ≥CIN2 and ≥CIN3 and high specificity to exclude ≥CIN2 in SurePath screening samples, and it has demonstrated noninferiority to the standard comparator test (GP-EIA) in this LBC medium. The extended genotyping design allows for detailed information on the presence of HPV types, including but not limited to HPV16 and -18; the precision of genotyping detection was found to be on a par with that of the GP-LMNX system. Furthermore, the Onclarity test can safely be repeated for quality control or assurance purposes even after prolonged storage of samples. The results from this study confirm that that the Onclarity test can be applied in primary cervical cancer screening using the SurePath collection medium.
